# Giant ectopic liver, hepatocellular carcinoma and pachydermia-a rare genetic syndrome?

**DOI:** 10.1186/1746-1596-6-75

**Published:** 2011-08-10

**Authors:** Matthias Dettmer, Peter Itin, Peter Miny, Manoj Gandhi, Gieri Cathomas, Niels Willi

**Affiliations:** 1 Cantonal Institute of Pathology, Liestal, Switzerland; 2 Department of Pathology and Laboratory Medicine, University of Pittsburgh, Pittsburgh, USA; 3 Department of Dermatology, University of Basel, Switzerland; 4 Division of Medical Genetics, University Children's Hospital, Basel, Switzerland

**Keywords:** ectopic liver, hepatocellular carcinoma, metastasis, autopsy, genetic syndrome, pachydermoperiostosis

## Abstract

Ectopic liver is a very uncommon developmental anomaly that predisposes to the development of hepatocellular carcinoma. We describe the second documented case of a hepatocellular carcinoma developing in the primary liver of a patient with a rare and uncharacterized genetic symptom complex. Also present was the largest ectopic liver ever reported, measuring 12 cm in diameter which contained a solitary focus of metastatic hepatocellular carcinoma. The primary hepatocellular carcinoma is believed to have arisen in the native liver from a hepatic adenoma that was diagnosed 15 years earlier. The patient's uncharacterised condition featured prominent thick, yellow skin over the dorsum of the fingers, and was associated with follicular hyperkeratosis, abnormal plantar creases, digital clubbing, misshaped ears, a lingua plicata and an angioleiomyolipoma of the right kidney.

This unique case of hepatocellular carcinoma arising from liver cell adenoma in a patient with an uncharacterised condition featuring a large ectopic liver invites discussion of the role of local factors in carcinogenesis in the parent liver but not the ectopic liver. It also underlines the imperative ongoing need for clinical autopsies.

## Background

Ectopic liver (hepar succenturiatum) is a rare developmental anomaly. The incidence was reported as 0.23% in a series of 5500 autopsies in 1940 [1], but probably with a more thorough work-up nowadays, the laparoscopic incidence varies between 0.47% and 0.7% [2,3].

Heterotopic liver tissue may be classified either as accessory liver, when it is connected with a thin stalk to the main liver or as a true ectopic liver when no such relationship can be established [4]. Most often, ectopic livers are found on the serosal surface of the gallbladder. In general, the reported sizes of ectopic livers range from a few millimeters up to several centimetres [2,5].

Most ectopic livers are clinically silent. In some cases, recurrent abdominal pain caused by torsion or intraperitoneal bleeding have been reported [2]. Ectopic liver tissue usually show normal histological architecture and are subject to the same risk factors and pathological processes as native liver tissue [4]. Fatty infiltration and alpha-1-antitrypsin deficiency have been described in ectopic liver [4,6]. However, the development of a hepatocellular carcinoma (HCC) in the ectopic liver is of utmost significance [7]. In fact, HCC can be observed in about 30% of ectopic livers [7]. The high incidence of neoplastic change in ectopic livers is explained probably by a different functional architecture with incomplete vascular and/or ductal systems. This results in longer exposure of ectopic liver tissues to carcinogenic substances thereby propelling its malignant transformation [8]. The reverse situation, wherein a HCC develops in the parent liver but not in the ectopic liver is exceedingly rare. Here, we present a detailed report of such a case in which the patient developed malignant change in the native liver but not in the ectopic liver.

Pachydermoperiostosis is a rare type of genodermatosis, characterized by pachydermia, digital clubbing and periostosis [9]. Recently, it has been mapped to chromosome 4q33-q34 and mutations in gene that encodes for 15-hydroxyprostaglandin dehydrogenase (*HPGD*). HPGD is the main enzyme required for prostaglandin degradation, including prostaglandin E2 [10,11]. The biology of prostaglandin E2 fits well together with the various clinical manifestations of pachydermoperiostosis. These include skeletal manifestations periosteal bone formation (periostosis) and resorption (acro-osteolyses) which result from the stimulation of osteoblasts and osteoclasts respectively; and finger clubbing due to prolonged vasodilatation [11]. Other factors such as receptor activator of nuclear factor (NF)- _k_B ligand or growth factors like Interleukin (IL)-6 are involved in the disease [12].

### Case Report

**Figure 1:**
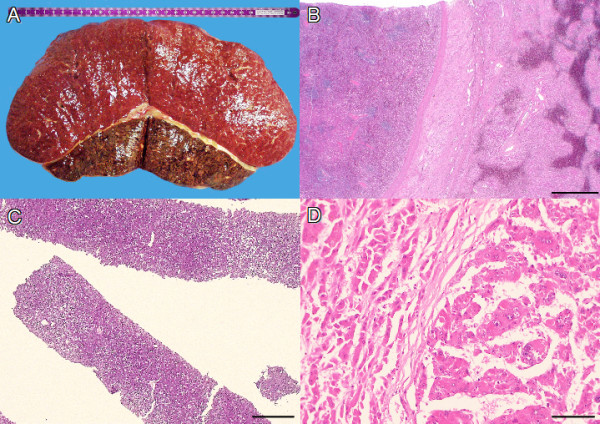
**Macroscopic and microscopic images**. A: macroscopic image of the ectopic liver, fused with the spleen. B: microscopic image of the ectopic liver (right), fused with the spleen (left). The organs are separated by the splenic capsule and loose connective tissue (12.5×, metering bar = 200 μm). C: microscopic image of the liver biopsy showing mild hepatocellular adenomatosis (10×, metering bar = 200 μm). D: microscopic image of the HCC (right) and the parent adenoma (left). The broad trabeculae and increased nuclear pleomorphism can be easily appreciated in the HCC (200×, metering bar = 100 μm).

A thirty year old Caucasian woman was treated for oligomenorrhoea with Norethisteron for six months. During follow up, a thorough clinical workup for hirsutism, chronic pruritus and abdominal discomfort revealed several nodules in both lobes of the parent liver, and an 18 cm large presumed splenomegaly (Figure 1A+B), both were diagnosed by a computed axial tomography (CT scan), the parent liver received an additional angiography. Significant portal vein hypertension was also present with signs of a portal vein occlusion. Subsequent explorative laparoscopy revealed three liver masses ranging from 1 cm to 6 cm. Liver biopsy was performed on the nodules and the diagnosis of a hepatocellular adenomatosis was established. The levels of testosterone, dehydroepiandrosterone and 17-hydroxyprogesterone were normal as were other routine liver function tests such as bilirubin, serology for hepatitis viruses, alpha-fetoprotein and carcinoembryonic antigen. No further therapeutic steps were taken. Nineteen months later, the patient developed spontaneous bacterial peritonitis. Explorative laparatomy did not reveal any source of infection. Ultrasonography showed a non-homogenous liver without any well defined mass. A repeat biopsy confirmed the previous diagnosis of hepatocellular adenomatosis (Figure 1C). Fifteen years later, the patient, who also had depressive psychological disorder, complained again of abdominal discomfort and progressive dyspnea. Progression of size and confluence of the liver nodules was determined along with increasing liver insufficiency. A biopsy of the hepatic nodules was performed again and, this time, hepatocellular carcinoma was diagnosed (Figure 1D). Liver transplantation was not considered due to suspicion of femoral bone metastasis revealed via CT-scan. The patient died after rapid deterioration of her general condition and liver failure, 15 years after her initial presentation.

### Autopsy

**Figure 2:**
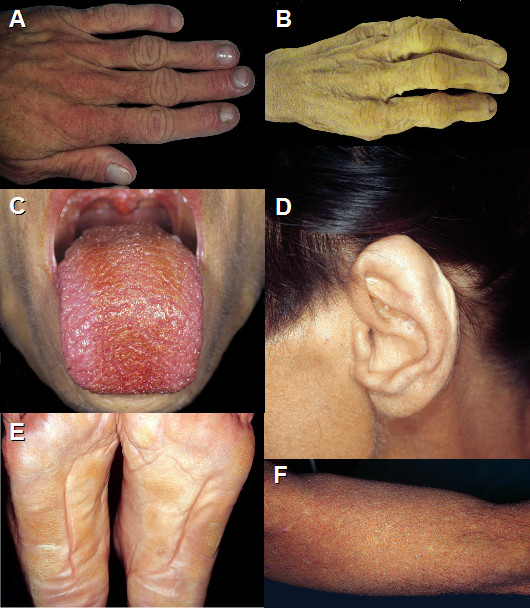
**Legends to clinical figures**. A: Mild digital clubbing. B: Pachydermic skin with marked folds on the fingers. C: Lingua plicata D: Ear malformation. E: Abnormal creases on the plantar surface of the feet. F: Follicular hyperkeratosis.

External examination of the body revealed a severe icterus, hirsutism and a malformed left auricle. Additionally, a striking hyperkeratosis, mild acanthosis but no parakeratosis was present on the skin of the soles of the feet. The skin and shape of the fingers was reptile like with mild digital clubbing. There were abnormal skin creases on the palms and soles (Figure 2). Examination of the internal organs revealed the following abnormalities: A lingua plicata, an enlarged liver that contained a 17 cm tumor with several satellite nodules. The residual liver tissue did not show cirrhosis. The spleen was enlarged and weighed 750 g. Cross section of the spleen revealed an ectopic liver measuring 12 cm in diameter and was attached to the spleen (Figure 1A and 1B). A stalk to the liver was absent and no efferent bile duct system could be identified.

After histological examination, the diagnosis of a dedifferentiated hepatocellular carcinoma (HCC) was established in the parent liver (Figure 1D). The tumour showed central vein invasion and widespread necrosis. Multiple well differentiated hepatocellular nodules with mild atypia, invading the surrounding liver, were found in the periphery of the principal HCC. The residual liver parenchyma showed cholestasis, little portal fibrosis, centrilobular sinusoidal dilatation and moderate centrilobular necrosis which was attributed to passive vascular congestion due to right heart failure. Multiple pulmonary tumour metastases were also present. The anticipated metastasis in the right femur which was believed to be responsible for the rejection of liver transplantation was in fact an enchondroma (diameter 2 cm).

The ectopic liver was separated by a fibrous capsule from the adherent spleen. The liver architecture was well preserved (Figure 1A and 1B). A metastasis of the primary HCC (0.9 cm) was noted. There were large portal tracts that contained branches of peripheral nerves. Moderate centrilobular necrosis similar to that seen in the native liver was also present. Minimal canalicular cholestasis was seen around the necrotic foci. Moderate centrilobular pericellular fibrosis was present. The large efferent bile duct was absent. Little orcein positive granular material was detected in the cytoplasm of periportal hepatocytes, indicating chronic cholestasis. The liver veins showed intimal fibrosis and were focally recanalized in the vicinity of a 1.2 cm scar.

Immunohistochemistry for androgen, oestrogen and progesterone receptors was consistently negative in the HCC as well as the parenchyma of the parent liver and the ectopic liver.

The right kidney harboured a lipoleiomyoma (diameter 0.8 cm). The hypophysis and adrenal glands were normal in size while both ovaries were atrophic.

## Discussion

In this report, we present a rare case of a patient having dedifferentiated HCC in the primary liver along with metastatic lesions in the giant ectopic liver and the lungs (lymphangiosis carcinomatosa). To our knowledge, there is only one other case of a Japanese patient who presented with similar findings, cited in a meta-analysis by Arakawa [8].

The malignant transformation of HA's has been well established in the literature [13]. In fact the first case, in a 21 year old woman who was treated with oral contraceptives for two years, was reported as early as the seventies of the last century [14]. Another paper reported HCC originating at the site of a former HA that had regressed after oral contraceptives were discontinued [15].

In our case, the HA persisted in the native liver and eventually underwent malignant transformation. Interestingly, this probably overshadowed the malignancy in the giant ectopic liver. The ectopic liver harboured a 0.9 cm malignant nodule which was interpreted as a metastasis from the HCC in the primary liver. This conclusion was based on the fact that both the lesions shared the same histological characteristics and that, unlike the native liver, no predisposing conditions such as HA or liver cell dysplasia was present in the ectopic liver. The history of portal vein thrombosis and intimal fibrosis of the liver veins suggests an impairment of blood outflow. As far as the carcinogenic process is concerned, both, the ineffective bile reabsorption and the altered blood outflow might have been contributory factors.

Generally, ectopic livers are known to form nodules ranging from several millimeters to several centimeters in size [2,5]. To our knowledge, this is the largest ectopic liver ever reported. We were unable to demonstrate a major efferent bile duct system, a problem already encountered by others [16]. It has been postulated that the produced bile may be reabsorbed by the lymph- or bloodstream [16]. Nevertheless, it still remains unclear why secondary biliary changes do not evolve in absence of the efferent bile duct system. The presence of an ectopic liver was unknown prior to autopsy and the patient was diagnosed as having presumed splenomegaly. Hence the autopsy procedure was performed without special precautions to look for the ectopic liver and its anatomical systems prior to splenectomy. Thus, it is possible, that there was a very thin efferent bile duct directly draining into the bowel which was overlooked.

In addition, the patient presented with a very unusual condition. During her lifetime, the provisional diagnosis of pachydermoperiostosis had been introduced by a consultant dermatologist (P.I.) at the time of detection of the HCC. This rare familial genodermatosis can present variably with the following features such as digital clubbing, swelling of peri-articular tissue, osteoarthropathy, arthralgia and arthritis, congenital heart disease, delayed closure of the fontanels, pachydermia and seborrhoea [9,11,17]. An incomplete form of this condition has also been described [18].

In this case, the patient had digital clubbing, albeit not a very prominent feature. Additionally, she had reptile-like fingers. Other changes included abnormal creases on the hands and feet, thick yellow skin and disseminated follicular hyperkeratosis. It is believed, that the skin changes are due to an activated Wnt signaling pathway with increased dermal fibroblast functions [19]. Because these changes were present since young adulthood, they were considered as primary, and not secondary to the liver tumour. The bones of forearms and hands and the abnormal skin of the fingers could not be investigated at autopsy for aesthetic reasons. The histological findings of the non-affected skin were consistent with the diagnosis of a pachydermoperiostosis, although well established histological criteria for this rare disease are not available. Nuclear steroid hormone levels especially that of nuclear androgen have been observed to be elevated in pachydermoperiostotic skin [20]. Interestingly, altered androgen metabolism and androgen receptor activity might also lead to HCC [21,22]. This observation may be a link between hepatic tumours and the skin manifestation in this disease.

The patient also presented with idiopathic hirsutism which can probably be explained by the recently identified mutations in *HPGD *(4q33-q34) that encodes for 15-hydroxyprostaglandin dehydrogenase which is the main enzyme of prostaglandin degradation [11]. A common endocrinopathy was ruled out by normal serum levels of steroid hormones and their metabolites.

The patient also had a very distinct auricle malformation. This has not been described in pachydermoperiostosis. In fact, this malformation resembles the case with Auralcephalosyndactyly described by Kurczynski, or the case of long chromosome 4 syndrome, described by Lipson, although the other features of these reports do not fit into the puzzle [23,24]. While, the trisomy 8 syndrome has also ear malformations similar to this case, it also has plantar abnormalities that are absent in our case [25].

Lingua plicata has been linked to Down syndrome, Sjörgren syndrome, Melkersson-Rosenthal Syndrome, psoriasis and geographic tongue [26]. However, an association of any of these conditions with pachydermia has not been published [27].

A search in pubmed and OMIM for "heterotopic liver" or "ectopic liver", "pachydermia", "hyperkeratosis", "lingua plicata", "angiolyomyolipoma" and combinations thereof did not reveal any usable hits [28,29]. Therefore this case is unique with an unknown etiology.

The worldwide autopsy rates have declined for many years despite their proven positive impact on various aspects in daily modern medical practice [30]. This case highlights the role of clinical autopsies in current practice since the patient was refused a live-saving liver transplantation due to an anticipated metastasis in the femur which-despite modern radiology-turned out to be in fact an enchondroma.

## Conclusion

We report the extremely rare case of a HCC in the parent liver, metastasizing into an ectopic liver in a patient with an association of unique clinical features suggestive of pachydermoperiostosis. It encompasses external malformations, resembling pachydermoperiostosis but cannot be explained by this alone. The internal malformations include a giant ectopic liver, fused with the spleen, a lingua plicata, an enchondroma and an angioleiomyolipoma of the kidney. This association may be either coincidental or represent an undescribed rare genetic syndrome.

## Consent

Written informed consent could not be obtained from the patient. To our knowledge, there are no relatives or next of kin available.

## Competing interests

The authors declare that they have no competing interests.

## Authors' contributions

All authors contributed to this article. All authors read and approved the final manuscript.
